# In vitro ion adsorption and cytocompatibility of dicalcium phosphate ceramics

**DOI:** 10.1186/s40824-017-0096-4

**Published:** 2017-06-08

**Authors:** Martha Schamel, Jake E. Barralet, Jürgen Groll, Uwe Gbureck

**Affiliations:** 1 Department of Functional Materials in Medicine and Dentistry, University of Würzburg, 97070 Würzburg, Germany; 2 Department of Surgery, Faculty of Medicine, Faculty of Dentistry, McGill University, Montreal, Quebec H3A 2B2 Canada

**Keywords:** Brushite, Monetite, Cell culture, Ion adsorption

## Abstract

**Background:**

*In vitro* cell testing of degradable bioceramics such as brushite or monetite is often challenging due to the ion release into or adsorption from the culture medium. These ionic changes are then mostly responsible for cell proliferation and activity, which prohibits the investigation of effects originating from surface topography or further material modifications.

**Methods:**

Here, we aimed to solve this problem by developing a pre-conditioning regime following the repeated immersion of brushite and monetite samples in various Ca^2+^, Mg^2+^ and PO_4_^3−^ containing electrolytes, followed by studying ion adsorption / release as well as changes in phase composition and in vitro cytocompatibility with MG63 cells.

**Results:**

The results demonstrated that by using DMEM cell culture medium in a ratio of 10 ml/sample was sufficient to minimize changes of ionic composition after 7 d with a daily change of the medium. This leads to changes of the surface composition with dissolution of the brushite phase. In turn, this also positively influences the *in vitro* cytocompatibility with a 2–3 fold higher cell number and cell activity on the DMEM pretreated surfaces.

**Conclusions:**

Controlled sample washing prior to cell testing using DMEM medium seems to be a valuable procedure not only to stabilize the pH during cell culture but also to maintain ion concentrations within a cell friendly range.

## Background

Calcium phosphate cements (CPC) are of high clinical interest for bone replacement due to their well-known biocompatibility in vivo [1–3]. Although a diversity of formulations are possible there are only two main products of the cement dissolution–precipitation reaction. Under neutral conditions hydroxyapatite (Ca_10_(PO_4_)_6_(OH)_2_, HA) is formed and under acid conditions orthophosphate is protonated and secondary phosphates such as brushite (CaHPO_4_•2H_2_O) or monetite (CaHPO_4_) are the main products of cement setting [4–6]. Whilst brushite is commonly formed due to kinetic reasons, monetite is only precipitated under highly acidic pH conditions, a water-deficient environment or by adding metal ions disrupting brushite crystal growth [7, 8]. Compared to HA cements, secondary phosphates have the advantage of a higher solubility under physiological conditions, which results in a faster resorption and bone remodeling in vivo [9]. In comparison to brushite, monetite shows a lower solubility under physiological conditions, but resorbs faster *in vivo* since monetite does not transform into low soluble HA at physiological pH [10].

Although brushite and monetite based cements show promising results *in vivo* [11–14], their in vitro characterization is challenging due to their metastable behavior under cell culture conditions. According to our experience this leads to a high release of phosphate ions into and an uptake of calcium and magnesium ions from the culture medium, whereas both effects have a detrimental effect on the cytocompatibility of the materials. This is problematic for the development of material modifications based on brushite or monetite (e.g. by adding bioactive metal ions), since it is then unclear whether observed effects during cell culture are correlated with the modification itself (e.g. by the released metal ion) or with the properties of the matrix and their capacity to change the ionic composition of the culture medium. The same would appear for an influence of surface topography on *in vitro* cellular behavior [15]. Recently, we were able to show that silica modified brushite cements showed an improved cytocompatibility compared to pure brushite samples [16]. However, this behavior was not correlated to the release of silicate ions, but to an altered dissolution profile of brushite during the *in vitro* experiments.

A solution to this problem might be a pre-conditioning of the samples prior to cell testing by immersion in calcium and magnesium containing solutions. This is thought to both remove unreacted, acidic cement raw materials, as well as to saturate the samples with calcium and magnesium ions. Here we studied in a systematic way the effect of such a washing regime of brushite and monetite samples prior to cell testing. Both changes of culture medium composition as well as the phase composition of the ceramic surface were correlated to the *in vitro* cytocompatibility determined with an osteoblastic cell line.

## Methods

### Materials

β-Tricalcium phosphate (ß-TCP) was prepared by sintering monetite powder (CaHPO_4_, Baker, Germany) and calcium carbonate (CaCO_3_, Merck, Germany) in a molar ratio of 2:1 for 5 h at 1050 °C. The sintered cakes were manually crushed with mortar and pestle and then sieved with 355 μm pore size-mesh prior to milling in a planetary ball mill (Retsch, Haan, Germany) for 60 min at 200 rpm. Cement powders were produced by mixing β-TCP powder in an equimolar ratio with monocalcium phosphate anhydrous (Ca(H_2_PO_4_)_2_, MCPA, Aldrich, Steinheim, Germany) in a coffee grinder for 30 s. Cement pastes were prepared by mixing the powder with water at powder to liquid ratios of 1.0, 2.0 and 3.0 g/ml. The pastes were transferred into silicone rubber molds (d = 15 mm, h = 2 mm) and set for 24 h at 37 °C at a humidity >90%. This resulted in a quantitative conversion of the cement powder into brushite according to XRD analysis. Monetite samples were prepared accordingly followed by autoclaving the samples at 121 °C for 20 min. All samples were sterilized prior to the following experiments by soaking in 70% ethanol followed by drying under sterile conditions.

### Methods

**Fig. 1:**
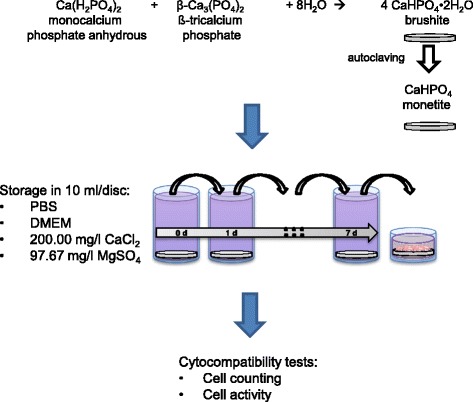
Preparation regime for brushite and monetite samples followed by preconditioning in different media and cell testing

Cements were either used without any washing regime (untreated reference) or they were stored in 10 ml 200 mg/l CaCl_2_ respectively 97.67 mg/l MgSO_4_ for 7d. These concentrations were chosen according to the Mg^2+^ and Ca^2+^ content of DMEM medium. For the DMEM group (DMEM: Dulbecco’s Modified Eagle’s Medium, Invitrogen Life Technologies, Karlsruhe, Germany) each cement disk was stored in 10 ml DMEM for 7d with a daily change of the medium (Fig. 1). Additionally, one group was washed 8 times for 2 h in distilled water (10 ml/disk) and afterwards immersed in phosphate buffered saline (PBS) for 7 d.

### Analysis

Porosity characteristics of the samples were measured by mercury (Hg) porosimetry (PASCAL 140/440, Porotec GmbH, Hofheim, Germany). Specific surface area was determined by nitrogen adsorption (BET-method, Autosorb-iQ-AG, Quantachrome, Odelzhausen, Germany). The ionic composition of each medium was analyzed by inductively coupled plasma mass spectroscopy (ICP-MS, Varian, Australia) against standard solutions of 10 ppm Ca^2+^, Mg^2+^ or PO_4_^3−^ (Merck, Darmstadt, Germany). The phase composition of the samples was determined using X-ray diffraction (XRD) analysis with monochromatic CuKα radiation (D5005, Siemens, Karlsruhe, Germany) in a 2θ range from 20 to 40 ° with a step size of 0.02 °. This was performed on both finely ground samples (for changes of the bulk volume) as well as on intact samples to investigate the influence of the immersion regime on the surface composition. Qualitative assessment of the diffraction patterns occurred via JCPDS reference patterns for brushite (PDF Ref. 09–0077), monetite (PDF Ref. 09–0080) and β-TCP (PDF Ref. 09–0169).

*In vitro* cytocompatibility testing was performed using the osteoblastic cell line MG 63 (ATCC no. CRL-1427, Rockville, MD). Cells were cultured at 37 °C and 5% CO_2_ in DMEM medium supplemented with 10% fetal calf serum, 100 U/ml penicillin and 100 mg/ml streptomycin (all from Invitrogen Life Technologies). Cells were cultivated on polystyrene (PS) as well as on unwashed and pre-conditioned brushite and monetite specimen. Samples were placed in quadruplicate into the wells of a 24-well plate and covered with cell suspension. Cytocompatibility tests were performed by measuring cell proliferation as well as cell activity after 2 days in culture on all surfaces. Cell counting was performed using a CASY 1 TTC cell analyzer (Schärfe System, Reutlingen, Germany). Cell viability was analyzed using the cell proliferation reagent WST 1 (Roche Diagnostics, Mannheim, Germany), whereas after incubating the cells for 30 min with the a 1:10 dilution of the WST reagent in DMEM at 37 °C, the absorption of the supernatant was photometrically quantified (Tecan, Crailsheim, Germany) at 450 nm. For each method and sample four readings were recorded and the mean values and standard deviations were calculated.

## Results and discussion

**Fig. 2:**
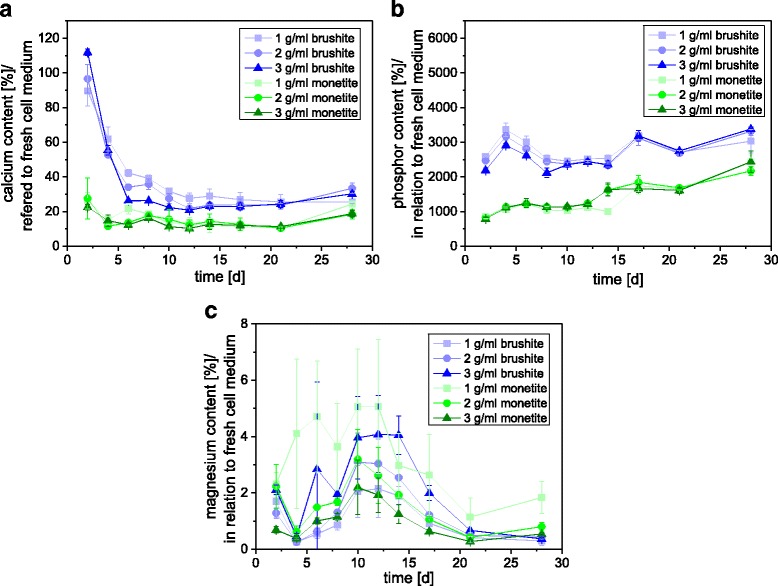
**a** Calcium, **b** phosphate and **c** magnesium ion release of brushite and monetite cement samples into DMEM medium over 4 weeks. Cements were produced with a PLR of 1, 2 and 3 g/ml

In a previous study [16] we observed, that calcium and magnesium ion adsorption and the phosphate release of brushite cement seems to be a crucial factor for cell response for secondary calcium phosphate ceramics. Therefore, we initially analyzed the ion concentration under cell test conditions (1 ml medium / sample) over a time course of 4 weeks (Fig. 2). As materials we have chosen pure brushite formed by a cement setting reaction as well as the anhydride monetite, which was obtained by autoclaving brushite. Due to the different powder to liquid ratios, the porosity / specific surface area varied between 30 and 63% / 1.5–2.1 m^2^/g (brushite) and 50–73% / 1.6–1.8 m^2^/g (monetite) (Table 1). The higher porosity values and the decreasing specific surface area for monetite can be explained by the higher density of monetite crystals (~ 2.92 g/ml) compared to the hydrated form brushite (~ 2.27 g/ml) [17]. The fact that only minor variations were found for the specific surface area during the transformation from brushite to monetite is related to the autoclaving regime. Here, the dehydration occurs via the liquid phase such that compact monetite crystals are produced, while a dry heat dehydration regime of brushite would produce highly porous monetite crystals with specific surface areas of ~20 m^2^/g [18].

**Table 1:** Porosity and specific surface area of brushite and monetite produced with a PLR of 1, 2 and 3 g/ml

Material	Porosity [%]	Surface area [m^2^/g]
1 g/ml brushite	63.5	2.14 ± 0.03
2 g/ml brushite	38.1	1.95 ± 0.23
3 g/ml brushite	29.9	1.54 ± 0.14
1 g/ml monetite	73.4	1.86 ± 0.15
2 g/ml monetite	56.5	1.71 ± 0.24
3 g/ml monetite	50.6	1.62 ± 0.20

The results of the initial immersion study showed an even increasing release of phosphate, which raised the phosphate content by the factor of 20–35 (brushite) and the factor of 8–20 (monetite) compared to fresh medium. At the same time, the cements adsorbed most calcium (monetite >80%, brushite ~60–75% after more than 5 days) and magnesium ions (>95% for both matrices). Monetite generally adsorbed more Ca^2+^ and released less PO_4_^3−^, whereas no clear difference was found for Mg^2+^.

**Fig. 3:**
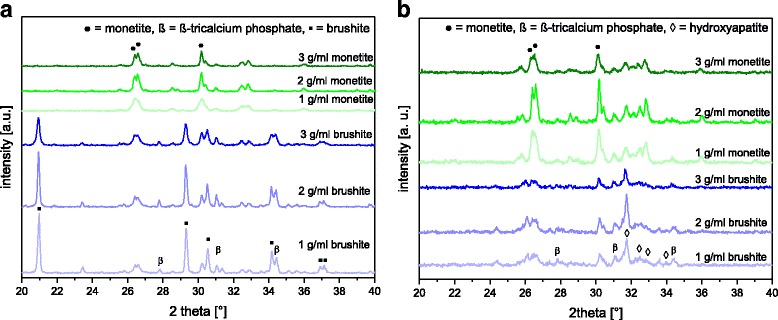
XRD patterns of brushite and monetite produced with a PLR of 1, 2 and 3 g/ml **a** before and **b** after 4 weeks in DMEM

This long term immersion regime also influenced the phase composition of the samples (Fig. 3), whereas especially brushite was susceptible to a complete conversion into the anhydride monetite and also partially converted into hydroxyapatite. Brushite is well known to transform into different more stable phases in vitro under neutral pH conditions, e.g. calcium deficient hydroxyapatite, carbonated hydroxyapatite or whitlockite [19, 20]. This effect was more pronounced for higher porous brushite, which enables a better fluid exchange between the culture medium and the cement bulk. This is important since HA has a higher Ca:P ratio (1.5–1.67) compared to brushite such that additional calcium ions are necessary, which have to diffuse into the pores to achieve a conversion even within the bulk of the matrix. Indeed, this was confirmed in an *in vivo* model, where it was demonstrated that low porous brushite was stable even over a period of 10 months in the femur of sheep, whereas at higher porosity a quantitative conversion into octacalcium phosphate and hydroxyapatite was observed [21].

**Fig. 4:**
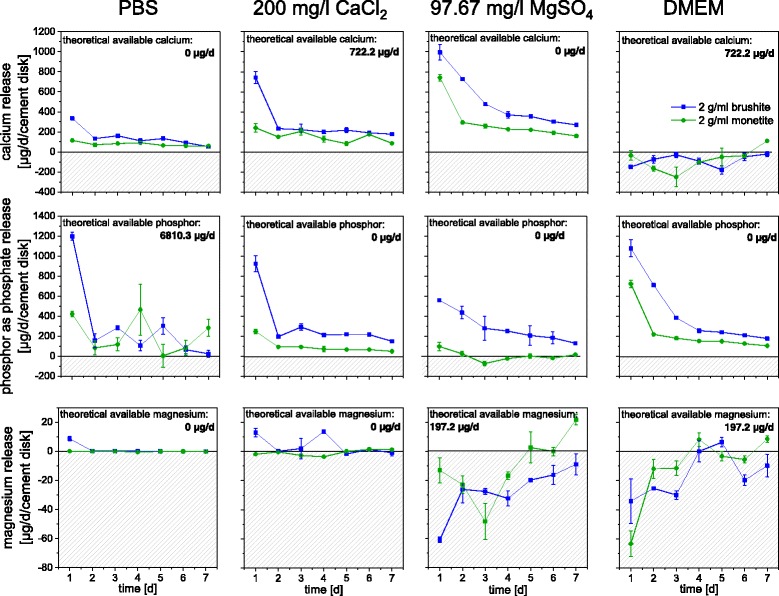
Ion adsorption during pre-conditioning regime using 10 ml solution per sample (PLR = 2 g/ml) with a daily change of the medium. *Shaded areas* correspond to ion adsorption from the media while the *white area* in the graphs correspond to ion release

**Fig. 5:**
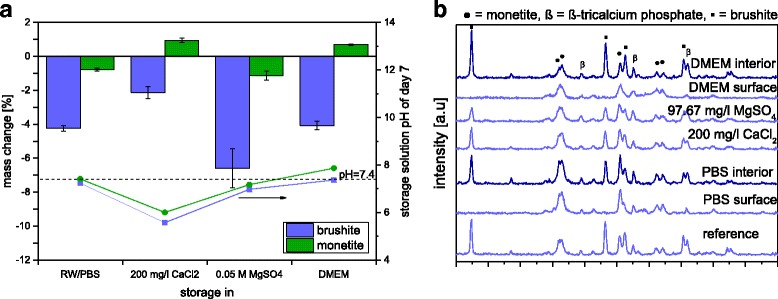
**a** Mass change of brushite and monetite (PLR = 2 g/ml) in 200 mg/l CaCl_2_, 97.67 mg/l MgSO_4,_ DMEM medium and PBS after 7 days and the pH of these solutions after the 7th day. **b** X-ray diffraction pattern of the brushite samples, either taken from the whole bulk volume after crushing with mortar and pestle (interior) or from the surface by directly placing the cement disc in the XRD sample holder reference (= untreated sample)

Since the observed changes in ion concentration of the cell culture medium as well as phase changes of the cement bulk can influence the outcome of in vitro cell culture experiments, the influence of a pre-conditioning regime on the above mentioned parameters was studied. This was performed by cement immersion in Ca^2+^ and Mg^2+^ containing solutions over a course of 7 days. Here, a ten times higher ratio between the volumes of immersion medium and cement sample was chosen compared to cell culture conditions. This was done to minimize saturation effects in terms of ion release (phosphate) as well as to provide a high amount of Ca^2+^ and Mg^2+^ ions to adsorb to the cement discs. This indeed reduced ionic changes of the different media after a course of 7 days (Fig. 4). Especially the immersion in DMEM medium was successful in terms of strongly reducing the phosphate release from the samples to a range lower than 100 μg/sample (~10 mg/l) after 7 days. High phosphate release exceeding approx. 15 times the normal culture medium concentration are known to be responsible for cell apoptosis under *in vitro* conditions [16]. In addition, a saturation of the samples with calcium and magnesium ions was achieved for DMEM medium. While there was only a marginal weight change of ~1% for the monetite samples after 7 d, brushite lost up to 2–6.5 wt.% mass (Fig. 5a). This weight loss is mostly a result of brushite dissolution from the surface of the samples as indicated by XRD (Fig. 5b) rather than from the bulk volume. This indicates that the ion adsorption and release is limited to the outer surface of the samples and is not diffusion controlled. Monetite samples did not show any change in phase composition after 7 d (data not shown).

**Fig. 6:**
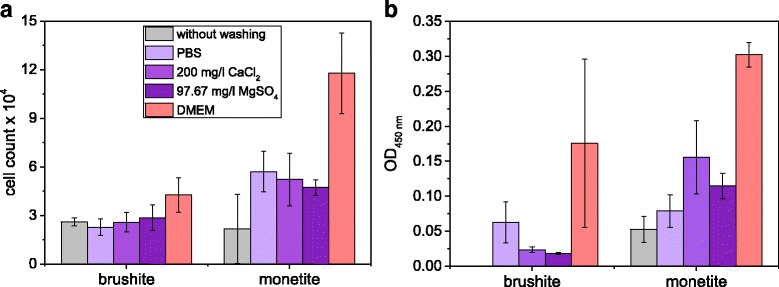
**a** Cell count and **b** cell activity according to WST-1 test of MG63 cultivated on brushite and monetite (PLR 2 g/ml) for 2 days, which were pre-conditioned with either 200 mg/l CaCl_2_, 97.67 mg/l MgSO_4_, DMEM medium or PBS over a course of 7 days

In a final experiment, an osteoblast cell line (MG63) was cultured on the surface of the pre-conditioned samples. Here, both cell number and cell activity according to the WST-1 test were increasing (Fig. 6), whereas the effect was more pronounced on monetite samples with a ~ 5 times higher proliferation rate and cell activity for DMEM treatment compared to the reference. Even this short-term biological experiment clearly demonstrated the necessity of pre-treating bioceramic samples prior to cell testing. Although this is likely done within most of the studies, the quality check for sufficient washing is mostly considered to be a stable pH after washing. Only few studies also addressed changes of the ionic composition of the culture medium as a responsible parameter for the outcome of the experiments [22, 23]. This applies not only for secondary phosphates as in the current study, but also to low soluble nanocrystalline hydroxyapatite ceramics as shown by Gustavsson et al. [24–26]. The latter has - due to the high specific surface area - a strong affinity to a broad range of mono- and divalent cations [27, 28], which leads to a non-linear adsorption of calcium (50% adsorption) and potassium (8%) ions from cell culture media [26]. This in turn has a strong effect especially for bone forming cells, whereby a Ca^2+^ depletion of the medium is known to dramatically decrease osteoblast proliferation and differentiation [29, 30].

## Conclusion

In this study we analyzed the influence of the washing procedure of the metastable dicalcium phosphates brushite and monetite prior to in vitro studies. It is postulated, that monetite shows a better cell responses attributed to the lower solubility and thereby phosphate release of this phase [9]. Due to the higher solubility of brushite it is completely washed out by PBS and DMEM, which resulted in a monetite surface and better cell response. Additionally, the saturation of magnesium and calcium had a beneficial effect for the cytocompatibility, as these essential nutrients are not adsorbed by the cement monolith anymore. The results clearly underline the need for controlled sample washing prior to cell testing. Here, the use of a large excess of serum free cell culture medium combined with multiple changing steps seems to be valuable to maintain ion concentrations within a cell friendly range. This will help to overcome the often observed discrepancy between the good *in vivo* results of calcium phosphate bioceramics (and their long term successful clinical use) and the strongly reduced cell growth on the ceramic surface under *in vitro* conditions.

## Abbreviations

BET: Brunauer-Emmett-Teller
DMEM: Dulbecco’s modified eagle’s medium
HA: Hydroxyapatite
ICP-MS: Inductively coupled plasma – mass spectroscopy
JCPDS: Joint committee of powder diffraction standards
MCPA: Monocalcium phosphate anhydrous
PBS: Phosphate buffered saline
PS: Polystyrene
ß-TCP: ß-Tricalcium phosphate
XRD: X-ray diffraction
